# A qualitative investigation of the perceptions of female dog-bite victims and implications for the prevention of dog bites

**DOI:** 10.1016/j.jveb.2015.07.035

**Published:** 2015

**Authors:** Carri Westgarth, Francine Watkins

**Affiliations:** a Department of Epidemiology and Population Health, Institute of Infection and Global Health, Faculty of Health and Life Sciences, University of Liverpool, Leahurst Campus, Neston, Cheshire, UK; b School of Veterinary Science, Faculty of Health and Life Sciences, University of Liverpool, Neston, Cheshire, UK; c Institute for Risk and Uncertainty, University of Liverpool, Liverpool, UK; d Department of Public Health and Policy, Institute of Psychology, Health and Society, Faculty of Health and Life Sciences, University of Liverpool, Liverpool, UK

**Keywords:** animal bites, public health, qualitative research, risk/determinants, risk perception, dog

## Abstract

Preventing dog bites is an increasingly important public health and political issue with implications for both human and animal health and welfare. Expert opinion is that most bites are preventable. Intervention materials have been designed to educate people on how to assess the body language of dogs, evaluate risk, and take appropriate action. The effectiveness of this approach is rarely evaluated and the incidence of dog bites is thought to be increasing. Is the traditional approach to dog bite prevention working as well as it should? In this novel, small scale qualitative study, the perceptions of victims regarding their dog bite experience were explored in-depth. The study recruited 8 female participants who had been bitten by a dog in the past 5 years. In-depth, one-to-one interviews were conducted, transcribed, and analyzed using thematic analysis. The findings indicate that dog bites may not be as easily preventable as previously presumed, and that education about dog body language may not prevent some types of dog bites. The reasons participants were bitten were multifaceted and complex. In some cases, there was no interaction with the dog before the bite so there was no opportunity to assess the situation and modify behavior around the dog accordingly. Identifying who was to blame, and had responsibility for preventing the bite, was straightforward for the participants in hindsight. Those bitten blamed themselves and/or the dog owner, but not the dog. Most participants already felt they had a theoretical knowledge that would allow them to recognize dog aggression before the dog bite, yet participants, especially those who worked regularly with dogs, routinely believed, “it would not happen to me.” We also identified an attitude that bites were “just one of those things,” which could also be a barrier prevention initiatives. Rather than being special to the human-canine relationship, the attitudes discovered mirror those found in other areas of injury prevention. A new approach to dog-bite prevention may now be required, drawing on other injury prevention strategies including awareness-raising and minimizing the damage caused by a bite when it happens.

## Introduction

Bites from companion dogs are political issues because high-profile media stories about dog bites capture the popular consciousness and spark highly emotive debate. Dog bites also represent a significant public health problem in the United Kingdom and other western countries, not least due to their costs to the health system (HESonline, 2012). Many bites are not significant enough to require medical attention and go unreported (Sacks et al., 1996). Dog aggression also causes considerable stress to the animal (Voith, 2009) and biting can lead to rehoming, relinquishment to an animal shelter, or euthanasia (Diesel et al., 2008, Mikkelsen and Lund, 1999).

Serious dog bites requiring hospital admission are reportedly increasing (BBC, 2011, Yee Hee Lee, 2014). Is our approach to dog-bite prevention not working as well as we think it should? Dog behavior experts focus their concerns on bites that are the result of dog aggression. The public health concern is for any type of dog “bites”, not all of which would be defined by experts as “aggression” e.g., play or predation (Lockwood, 1995). To a member of the public, any bite may be described as an “aggressive” action, adding further confusion. Most bites are considered by experts to be preventable (De Keuster et al., 2006, Mills and Levine, 2006), and most people are bitten by dogs familiar to them (Voith, 2009). The view is often presented that bites occur because people misinterpret or do not recognize fearful dog behavior (Overall and Love, 2001, Yin, 2011, doggonesafe.com, 2015), suggesting that bites due to “aggression” are one concern, so intervention programs have traditionally targeted the education of children and adults about signals that a dog is concerned and may bite (Wilson et al., 2003, Duperrex et al., 2009, Schwebel et al., 2012). However there is little research to evidence if, and how, this approach actually prevents bites.

The success of this educational interventional approach in promoting behavior change and preventing bites is dependent on a number of factors. First, quantitative evidence suggests that in 40% of dog bites the victim was not interacting with the dog before (Cornelissen and Hopster, 2010), so targeting victim behavior may not be appropriate in these cases. Second, success of the traditional intervention approach also depends on the context in which the dog bite occurred (play, predation, or aggression due to fear) and whether the bite occurred in a home or in a public place.

Third, there is an influence of the perceived level of threat in terms of severity and degree to which one is susceptible to this threat and the ability of a person to negate the harm. This includes effectiveness of the response in negating the harm (response efficacy) and capability to enact that response (self efficacy) (Peters et al., 2013). In short, education of potential victims (e.g., anyone who is ever near a dog) about fearful dog behavioral signs will only be effective in preventing bites if they believe that a dog bite is a severe enough threat to want to avoid, that the dog (which may be their dog) might bite them, that there is something that they can do (or not do) to effectively prevent the bite from occurring, and that they are able to change their behavior in that situation to prevent the bite from happening. Risk communication theory highlights how important it is to compare the opinions of experts with lay beliefs (Austin and Fischhoff, 2012), yet this approach has not been used in dog-bite research.

Despite research about risk factors for dog bites (Overall and Love, 2001, Newman, 2012), evidence has been inconclusive. This may be because dog bites that occur in different contexts may have different causal mechanisms. It may also be due to an over-simplified view of dog bites as having simple ‘causes’, whereas in reality there is likely a complex multifactoral series of events and circumstances that will all contribute to the likelihood of a dog bite occurring. Here it is possible to borrow from the socio-ecological systems perspective and apply it also to dog-human interaction events (for another e.g., see [Westgarth et al., 2014]. For example, a dog may have a genetic predisposition for reactivity, a lack of early social exposure, and pain due to a medical condition. The victim may be under the influence of alcohol and behaving erratically when approaching the dog. All may affect risk, and prevention strategies must address these multiple contributing factors.

An in-depth, qualitative perspective may provide fresh insight into this complexity. Qualitative methods are particularly suited for understanding social phenomena in natural settings and have been used to illustrate how people interpret and use health care messages (Pope and Mays, 1995). In-depth investigation using qualitative research methods can be used to investigate perceptions, interpretations, and experiences (Mason, 2002) across and between different dog bite contexts.

Previously published qualitative studies about the dog bite experience have been limited and have focused on particular aspects of the experience. Sanders (1994) studied reasons given by veterinarians for dog bites noting that dogs were often excused from blame because of the situation (e.g., the dog was in pain) or the relationship with their owner (as incapable of exercising appropriate control over the animal). Dog owners being defined as “good” if they attempted to control their animal or were able to give a prior warning (Sanders, 1994). Rajecki et al. (2007) discuss a single case study through the last day in the life of a Doberman. Despite biting the female owner 3 times, at no point is the dog described as a “bad” dog. Instead, the male owner explains the dog's increasing aggressive tendencies to “moodiness”.

In these studies dogs are almost unanimously viewed in a “positive” light (Rajecki et al., 2007) with the dog's behavior often viewed as the responsibility of the owner, or caused by external factors that are not the fault of the animal (Sanders, 1990, Sanders, 1994, Rajecki et al., 1998, Rajecki et al., 1999). These research studies do not address the multifactorial circumstances surrounding the dog bite. The focus of prevention regarding dog bites is often targeted at the owner or on victim behavior, rather than how injuries are most effectively prevented (Hemenway, 2013) even though it is widely known that interventions must address multiple factors and levels in an ecological perspective in order to be effective (Bond and Hauf, 2004). Thus it is appropriate to now investigate whether the focus on victim behavior around the dog is an effective mechanism for preventing dog bites.

The aims of our study were to 1) explore the victim perception of what constituted a dog bite; 2) explore how victims perceive the circumstances and events that led to them being bitten by a dog; 3) examine how the victim regards the dog bite experience in terms of prevention of future bites; and 4) to inform public health policy relating to dog bite prevention and treatment through discussion of findings in terms of the theoretical mechanisms of prevention.

We are aware that the retrospective views of the victim are only one part of the story and represent a particular perspective. In qualitative research we are not seeking an objective truth about a causal mechanism, but rather seek to understand the perceptions, beliefs, and experiences of the victim to provide context to bite events and inform the likely barriers to prevention.

## Materials and methods

Detailed one-to-one interviews allowed scope for the participant to tell their story in-depth and for the researcher to ask questions to understand the circumstances (Green and Thorogood, 2009a).

### Data collection

The intended sample was adults (aged 18 years or over), living in the Merseyside or Cheshire area, who had been bitten within the last 3 years by an owned dog. Eight participants were recruited and interviewed by CW (female), either in their home or at the University. All the participants were female, aged between 20-60 years, with education levels ranged from GCSE or O'level to graduate. Demographic data are described in Table 1. Participants were recruited via posters and leaflets advertising the study in veterinary surgeries, dog training establishments, community centers, shop notice boards, and social media sites.

Although memory and recall accuracy can be an issue over time, most of the interviews occurred within 1 year of the bite occurring, and 3 within a couple of months (Table 2), potentially increasing validity of the recall. It became apparent during one interview that the participant had actually been bitten 5 years ago. The issue of recall bias and memory is less of a concern in qualitative research due to the focus on victim beliefs and perceptions at the time of interview rather than on an accurate objective history of events.

A pretested interview schedule (Box 1) was used to guide the participant through the chronological events surrounding the bite experience and reflections on the bite incident. Participants had the opportunity to detail specific aspects of events that were significant to them. As participants were providing a retrospective account of their bites based on their own perspectives, this inevitably included significant reflection on the incident. Discussion points were repeated back for clarification and further elaboration. Participants were aware of the researcher's interest in the topic of dog bites and during the conversations personal dog ownership was sometimes discussed. Interviews lasted between 14-50 minutes and were audio recorded on 2 digital recording devices. Demographic data were collected immediately after the interview. Extensive notes were also made after each interview, detailing impressions, and significant discussion points.

### Data analysis

The recordings were fully transcribed and names of people and dogs changed. A thematic analysis framework was used (Green and Thorogood, 2009b) which provided sufficient theoretical freedom to analyze the wide range of themes that emerged from the data. This framework enabled the development of a detailed and complex account of the data representing the experiences of the participants involved (Braun and Clarke, 2006). Transcripts were coded line-by-line (Braun and Clarke, 2006) using Excel, then key elements of the respondents accounts were derived inductively into themes (Pope et al., 2000) by categorizing recurrent or common sentiments expressed. Key themes selected were those important to the research question and did not depend on occurrence in all the interviews (Braun and Clarke, 2006). The lead researcher played an active role in identifying and reporting the themes considered interesting and developed the thematic framework through which to interpret the data. She consciously reflected on her preconceptions to allow the data to speak independently, but felt her unique understanding of the research context due to a background in dog training was also beneficial to the interpretation (Strauss and Corbin, 1998).

## Results

### What is a dog bite?

Part of being able to prevent dog bites requires defining what we are trying to prevent. Understanding of what constitutes a dog bite varies considerably. Self-selection of participants who deemed themselves to have been bitten still resulted in significant variation in the perception of the severity of an injury required for a “bite” to have occurred. All participants reported that a bite involved contact between teeth and skin, and breaking the skin was unanimously agreed to be more serious than not. One participant did not consider a bite to have occurred if the skin was unbroken, but later contradicted herself by stating that the dog had previously bitten but did not break the skin then. A bite was often differentiated from a nip, which was deemed less serious. One participant felt that if the dog was playing, the bite probably would not break the skin, and others felt that any contact during play was not a “real bite”, because there was no intent to injure (i.e., there was an absence of any indication of “aggression”). Others, however, considered that a bite had occurred if the dog made contact with teeth, even in play, and if any damage at all occurred. Establishing consensus of what constitutes a bite is essential as educational interventions are mostly designed to address reading the signs of aggression of the dog before “a bite”.

### Description of the bite incident and why it happened

Table 2 describes the main bite incident, whether they were bitten by their own dog or another dog, the bite location, and whether they had also been bitten on a previous occasion.

For 6 of the 8 participants, the primary bite incident discussed was not their first. Two discussed being bitten by their own dog, 3 by dogs known fairly well to them (belonging to a family member, friend, or a regular grooming client), and 3 by dogs rarely (belonging to neighbors) or never seen before (a stranger in a park). Four participants were interacting directly with the dog previous to the bite, 1 was walking the dog and holding it by the lead thus indirectly interacting with the dog, 1 was in the vicinity of the dog but not interacting with it, and 2 did not even know that the dog was present until the bite occurred. For those bitten while interacting with the dog, the bites occurred while doing things that were considered by the participants to be normal and acceptable interactions with that dog (e.g., petting, grooming, speaking to it, walking the dog on a lead).

The bites discussed resulted in pain, puncture, and bruising, and 4 participants received medical attention including cleaning and dressing the wound, a tetanus vaccination, or a course of antibiotics.

### Don't blame the dog

The participants were asked whether they felt that anyone was to blame for the main bite incident discussed. None of the participants blamed the dog, and many felt the need to state this clearly. Any discussion of blame towards the dog was indirect in that the perception was that the dog was acting on natural instinct and just being an animal. Six of the 8 participants blamed themselves in some way and viewed it as their fault. Self-blame was a particular strong issue for 2 participants who perceived themselves as “experienced” with dogs, through extensive ownership and employment or voluntary work. These participants felt they were responsible and they should have behaved differently to prevent the bite.

*Yes, I'm to blame. I still hold that my reactions to his [dog] aggression are what caused the bite. In almost every circumstance that I have been bitten, I have been to blame. And I don't just say that because I love dogs (laughter) and I don't blame dogs for anything. […] But I can't think of any occasion that I've been bitten where I couldn't have handled it better.*

Barbara—bitten by dog that she knew well

*At the time, me and my sister [were to blame] because we should have been a bit more careful and a bit more sensible about it. I mean it could be one of those things that you could never know, I suppose any dog could bite you. But I don't really believe that. I think, because we were both experienced with dogs…that we should have known better. I did at the time think that maybe she should have been a bit more careful about who she was letting alone with the dog that she probably didn't know particularly well, but I don't really blame, I certainly don't blame the dog, at all.”*

Claire—bitten by family member's dog

If the dog was not the participant's own dog then there was a tendency for blame to transfer to the owner. For 2 participants, there was a view that both the victim and the owner had elements of blame (in the case of the dog with neurologic issues described in the quote previously by Claire, and in the case of the groomer Fran, as the owner had not brought the dog in for a while—see quote in Table 2). For 2 participants, Annie and Gina, there was a clear perception that the owner's inactions led to the bite and therefore it should have been their responsibility to take action to prevent the bite because they should have used their knowledge and experience of the dog to intervene.

*Well … they shouldn't have let the dog out. And they should have had gates up. So yes I do blame them all really.*

Annie—bitten by neighbor's dog

However, for 1 participant, the issue of blame was not clear, and it became clear that the level of responsibility of the owner was perceived to vary depending on their knowledge of the potential for an aggressive incident.

*It's difficult because it depends if the woman knew that her dog had tendencies to bite people. She looked pretty shocked so maybe she didn't, so in that case nobody's to blame. But if she, if it had happened before and she's still taking her dog around near people then maybe she is to blame.*

Ellie—bitten while running

Perceived responsibility of the owner in bite prevention varied depending on their level of knowledge and whether they perceived that they could have predicted the bite. This finding suggests that it may be difficult to expect owners to prevent bites if they are not aware in advance that it has a strong possibility of happening (until at least one bite occurs). The perceived level of responsibility of the owner also depends on who the dog bites (a stranger or someone familiar) making it difficult to predict in advance who will be to blame if a bite occurs. Likewise, whether the victim felt that they were responsible for what happened varied depending on how well the victim knew the dog, and how experienced they felt they were with dogs, rather than the actual circumstances of the bite event. A prime example is Barbara, experienced in rehabilitating aggressive dogs, who was bitten on her leg as a result of “redirected aggression” when a dog-aggressive dog she was walking for a friend was approached by another dog. She tightened the lead and the dog turned and bit her on the leg. Despite trying to prevent an incident, Barbara still strongly blamed herself and believed that if she had just done something different (such as turning around earlier and walking the other way) the bite would not have occurred. In summary, rather than it being a simple factual matter of who caused the bite (and consequently who could have prevented it), responsibility and blame are a construction made in hindsight based upon whom the victim is, their relationship with the dog, and the perceived knowledge levels of the owner and/or victim.

This theme of “Don't blame the dog” can be discussed in light of preventive theory. Attributing blame is associated with coping mechanisms (Bulman and Wortman, 1977). Self-blame can be part of regret and feelings of self-blame are constructed from having made a poor decision and the severity of the outcome (Connolly and Zeelenberg, 2002). Feelings of self-blame and regret were even higher when the participant considered themselves experienced with dogs, because they “should have known better” and thus made a very poor decision. Thus, within this theme, we also notice the influence of perceived efficacy—one's capability to enact a response that is effective in negating the harm (Peters et al., 2013). If perceived self-efficacy to enact a negating response was believed to be high in “experienced” people, then behavior change should have been likely, unless the perception of threat was perceived to be low. This brings us to our next theme.

### I didn't think it would happen to me

All the participants felt that they had knowledge about dogs, mainly from direct experience of dogs either from owning them or working with them, guidance from friends or family members, or from informal education as a child or an adult. Six of the 8 had also suffered previous bites. The participants felt that their fundamental knowledge and previous experience should have prepared them and enabled them to take action to avoid the bite. However, they did not avoid it. A common perception among the participants was that they “didn't think it would happen to me” so were unprepared to use this knowledge and experience to act and prevent the bite. One participant even expressed disbelief that the bite would actually happen even when the threat level began to increase.

*I: And then what happened … were you scared before he bit you?*

*G: A little bit nervous, because it was like two dogs jumping around there. But I still at that point didn't think I was going to get bitten. Not for a million, not in a, not for a moment did I think I was going to get bitten.*

*I. Why do you think that is?*

*G. I don't know. I suppose still, I still trust dogs, maybe.*

Gina—bitten delivering a parcel to neighbor's house

Even if the participant knew they were at risk with this dog there was still a belief that it wouldn't happen this time.

*I. Why did it upset you when your own dog bit you?*

*C. It was a shock. It was..well no it wasn't unexpected. It's because you kind of take it personally I think and it did bother me. We'd had him, it wasn't like he was new to us at that point, we'd had him quite a long time, but I knew better, from what I was doing I was, basically as he was asleep, I was reaching to grab the remote from near him and, I should have known better, and I knew he'd go for me but you kind of expect that when you've got that trust bond with your dogs that they maybe wouldn't, even if you do something to upset them, so that's probably why it upset me because I was maybe expecting him not to be like that even though I knew that he would kind of thing so. It's silly really… Any other dog you could probably do it with but with him it's just something that you can't do because, it was like a movement next to him and it startled him and as he was asleep and you can't do things like that with him and I knew I couldn't do things like that but, for whatever reason I did. I think because I was trying to do it as he was asleep so that he wouldn't be aware of it but he did and he went for me.*

Claire—describing being bitten by her own dog on a previous occasion

The perception that “It would not happen to me” is an example of the degree to which one believes they are susceptible to the perceived threat, which will therefore have an impact on how likely behavior change is to occur (Peters et al., 2013). When the danger of a dog bite occurring is considered low, behavior change is unlikely to occur. Even if participants had been bitten before they still held the belief that “it would not happen to me”. This makes it difficult to know how to target interventions. If there is no perceived need, there is no need to participate in education, or to act upon any new knowledge, because the person does not believe a bite will happen to them, or could happen again. They will have little regard or follow-through for interventions that educate about how to take preventative action. Therefore, their behavior will not change.

### Just one of those things

Another shared perception was that sometimes dog bites were “just one of those things” that happen when around dogs. In particular, in 2 of the bite contexts (the groomer and the owner bitten multiple times handling their dog), the participants were fully aware of the risk, but continued anyway.

*She didn't want me to do what I was doing. And she'd already told me not to. But I carried on.*

Debra—bitten on multiple occasions by own dog

*Attempting to bite really doesn't bother you, you know. You can cope with attempting to bite if you keep your eyes open and you know which ways, which strings to pull with certain dogs. But sometimes you do get caught off guard.*

Fran—dog groomer

This perception that dog bites are “just one of those things” fits with the predictions of preventive theory. When threat is high but efficacy low, defensive reactions will occur such as denying the severity of a threat (Peters et al., 2013). For the 2 participants that were in a high-threat situation (the groomer, and the owner who lived with a frequently aggressive dog), self-efficacy was felt to be low (they could not easily avoid interacting with the dogs in a potential bite context), and bites were felt to be just one of those things that you could sometimes avoid, but sometimes not. So even when the threat was high, self-efficacy was low, and participants responded with an attitude of it “doesn't bother them” (denying the severity of the threat), and accepted that bites happened and there was nothing they could really do about them.

An acceptance of the risk of a dog bite as a somewhat unavoidable outcome of being around dogs is a further potential barrier to prevention initiatives. If potential victims do not believe that dog bites can be avoided, then they will not identify their need to participate in educational prevention information. Neither will they believe they have the efficacy to change their behavior to avoid a bite when signs of a potential bite situation are recognized.

### Reflection on the bite: before and after the event

For 5 of the participants, a bite experience did make them, in the short term, consciously reflect on their behavior around dogs. For these participants, it was also only on reflection, rather than in the moment, that they felt that they should have read the situation better and therefore prevented the bite. After the event, participants supplemented their previous theoretical knowledge about the signs of dog aggression with new knowledge from their own experience. They used this combination of knowledge to begin to assess their potential risk of being around dogs in the future.

*It just makes me more aware of what people have said to me, you know when I was younger, you know you don't just go straight up to a dog and pat it on its head kind of thing.*

Debra

*It was a learning curve I suppose. It was the first time I began to think about how you handle an aggressive dog, and what you look for in a dog, and what signs you look for, I'd never had to do that before.*

Barbara

All participants used the experience of being bitten to develop their own strategies to assess the risk that dogs posed in the future, although some then did not go on to significantly change their behavior, as seen above. Gina reported being “less likely to trust a dog”; and Ellie “a bit more wary of dogs,” but none of the participants who had a dog, reported concerns about their own dog after being bitten by somebody else's dog. Although these strategies often did include increased “wariness” or “cautiousness” around dogs at least temporarily, this tended to be related to specific dogs, usually similar breeds or dogs that bit them or similar contexts to which the bite occurred, rather than generalizing to all dogs.

**I. If you could go back to the day of the dog bite, is there anything that you would change?**

*E. Be more aware that there's a dog next to me.*

*I. So you think it's made you more aware when you're out doing your exercises?*

*E. Yeah. I try to be, yeah. I don't know. I really don't know why it bit me, maybe just cause I was running past it, it just though “ooh, rabbit” (chuckles) I don't know it though, but I'll just try and be a bit more aware in the future.*

*I. Okay. And are you planning to do anything in the future to prevent yourself from being bitten again?*

*E. Not run next to a dog.*

Ellie—bitten while out running

*Just specifically to that dog really, I just didn't want to go near him and I was very wary of him […] Maybe if I came across a dog with similar problems to him, maybe I would see (Name) in that dog and I would be a bit more careful.*

Claire—bitten by family member's dog

*Well I wouldn't put the…I'll never ever put the magazine through the door again, I just leave it in the gate. I would never do that now.*

Annie—bitten by neighbor's dog

Most participants also expressed that they “still loved dogs” after being bitten (Barbara, Claire, Debra, Fran, Gina, Helen), and it was important to them that the bite did not affect their general view of dogs which would be upsetting to them (Claire). Wariness was even presented as “not logical” because “I know ninety-nine point nine recurring percent of dogs are fine, and are not going to bite” (Ellie). These participants did not really want to change their behavior around dogs and therefore if they wanted to be around dogs they accepted that being bitten was a potential outcome. This was particularly apparent for Fran the groomer.

*I. If you were bitten again in the future, do you think it would put you off your career or would you stay the same or?*

*F. I don't think I'd be put off by any bite.*

*I. No?*

*F. At this minute. I don't think so.*

*I. Why is that?*

*F. I think the only thing that would stop me is if it actually hurt me enough that I was unable to groom anymore. Just because it's something I love doing and am passionate about. And I don't think, think it would put me off whatsoever unless I was unable to do it.*

Fran—Groomer

At the time of the interview, neither of the owners bitten by their own dogs had sought expert behavioral advice, despite one of them having an established relationship with a behavioral expert through puppy class attendance. They also did not rehome the dog or change their behavior significantly with the dog. Other than being more “wary of what it might do”, the dog continued to be managed in a very similar way. The participant bitten while walking the dog and her friend who owned the dog sought behavioral advice together, after the dog also bit the owner twice in similar circumstances. No other owners sought behavioral advice, to the victims' knowledge.

One owner responded to the incident by providing a cover for the dog to avoid getting the dog in trouble or to avoid it being perceived as a “dangerous dog” (despite the fact that it could now be viewed as such, and could constitute a risk to the owner and others).

*I made up a story, that my mum's cat, who is fifteen and is really old, had chomped on me when I was stroking her […] because I think people think, dangerous dogs, you know, dog bites are all, the media's all over it […] And I just wouldn't want anybody to think “oh, she's got a savage dog, she's got a dangerous dog, she needs to get rid of that dog.”*

Helen—bitten on face by own dog

The reactions to the bite event appear to be affected by the victim's relationship with the dog and the victim's feelings towards dogs in general. These feelings may cloud judgment about future risk of being bitten as feelings towards dogs were often unchanged longer-term by the bite incident, and some of the participants had been bitten multiple times already. This is an example of truth bias—wanting to believe that dogs would not bite them even though they know they can. People in well-developed relationships are more likely to judge greater truthfulness from them (Stiff et al., 1992); thus, it is perhaps not surprising that those who consider themselves to have strong relationships with dogs expect the outcomes of their interactions to be positive.

In summary, the various contexts of dog-bite incidents and differing perceptions of the causes poses significant problems for public health practitioners when trying to establish standardized prevention tools. The varied perceptions of what constitutes a bite may affect how people interpret the events occurring in the lead up to the bite, whether they believe the dog “intended” to bite (for example, bites given in “play” not being “real bites”), or how severe a bite is interpreted to be (for example, “just a nip”). These differing perceptions of what actually constitutes a bite, the perception that “it won't happen to me,” the dismissal of it as “just one of those things,” and the failure to blame the dog all make it difficult to see how a “one size fits all” educational program would be able to provide the knowledge individuals need to recognize and assess the varied potentially dangerous situations they may find themselves in with dogs. These responses are also recognized barriers in the theory regarding enacting behavior change in the face of threat. Reactions to the bite and perceptions of responsibility and preventability appeared to be more grounded in individual relationships with dogs and the dog, and personal beliefs about animals, than what actually happened in the bite circumstance and how preventable it may have been.

## Discussion

### Strengths and limitations

This preliminary study begins to identify how experiences from victims of dog bites are essential in the development and the implementation of strategies to tackle dog bites. There is a clear need for further studies using participants with more varied backgrounds and demographics, including children and parents. Children represent a significant proportion of hospital presentation of dog bite victims (Sacks et al., 1996) and are often the subject of the most emotive media coverage. Our study was limited in that it was a convenience sample and the volunteers who came forward for the study were all women. It may be that women are more likely to volunteer and men may be harder to reach. Men may require active targeting in any future studies, which should also target dog-bite victims more directly via referral mechanisms. Events leading to children or men being bitten may be significantly different as may the reflection after the event. The stories retold by the participants in this study were retrospective rather than immediate postincident responses and later follow-up, which may be required to assess how the experiences and reflections change over time. The data revealed wide-ranging views and participants reported different dog-bite contexts, including those bitten by their own, familiar and unknown dogs, and none of the bites were deemed very serious. This is a limitation that requires future investigation, however most dog bites are thought to go unreported and be minor, as were these. Finally, our study only considered the perception of victims, which may be different from the view of the owner. Due to the preliminary nature of this research project, any recommendations are made with caution.

### Summary of findings and comparison to previous research

The findings of this study suggest that preventing dog bites is not as simple as often portrayed (e.g., Yin, 2011, doggonesafe.com, 2015). Current interventional approaches may be only targeting exceptional circumstances (Overall and Love, 2001). Although victims often blamed themselves in some way for what happened, the interviewer, with behavioral expertise, felt that realistically only one of these victims could have been expected to behave much differently given the situation. Two of the participants were put into risky situations due to their career or interests, and 2 were bitten doing what they considered to be routine activities with dogs that they knew well. Although bites were easily justified after the event, they were not deemed predictable to the participants before it. Three participants did not believe the situation to be high risk because they were not directly interacting with the dog (2 did not even know the dog was present until it bit them), a finding that concurs with previous evidence that most people bitten in a public place were not interacting with the dog (Cornelissen and Hopster, 2010). Many of the participants felt knowledgeable enough to behave appropriately around dogs and had acted in the same way previously with that dog with no problem. In each case, the result was that the participant was unable to adequately assess the situation and intervene to prevent the bite from happening. Although after bites participants felt that the experience had made them more aware of their actions around dogs, this perception did not appear to translate into a reduction of risk in reality, as many participants had been bitten on more than one occasion.

Our findings are supported by prevention theory and demonstrate barriers posed by low levels of perceived danger, low perceived self-efficacy to enact behavior change, and defensive reactions in denying severity or susceptibility of the threat (Peters et al., 2013). In such situations, the use of typical fear appeals that raise awareness of which dogs are likely to bite are unlikely to be effective.

The complex perspective participants had about the dog bite is unsurprising due to the generally complex nature of our relationships with companion animals (Sanders 1999), and the view of them as both civilised and ‘human’ but also animalistic and chaotic (Belk 1996). Our findings agree with previous studies suggesting that animals are less likely to be blamed or punished for their actions than are people (Rajecki et al., 1998, Rajecki et al., 2007), and that bites are a result of external factors (Rajecki, Rasmussen et al., 1999). These views may cloud how we tackle the prevention of injuries associated with animals in comparison to other causes.

The perception that individuals did not feel at risk of being bitten until it happened may be the most significant barrier to educational dog-bite prevention initiatives. If the aim of educational strategies is to increase knowledge to assess risk and therefore change behavior, this will only work if the potential victims are adequately able to assess a situation and implement a strategy during an incident that could result in a bite. Based on the experiences from our participants, their mostly positive interactions with dogs resulted in their trusting of dogs and this may override the belief that the dog might bite them and result in a perceived negligible risk. This also fits with previous evidence that people feel that their own dog is least likely to bite them (Moss and Wright, 1987), despite evidence showing that people are more likely to be bitten by dogs familiar to them such as their own dogs (Voith, 2009).

Our key themes identified surrounding prevention clearly match with 3 common beliefs recognized as impediments to other injury prevention “it will never happen to me,” “accidents happen,” and “victim blaming” (Hemenway, 2013). Although participants felt that the dog bite was unavoidable, or the victim or owner's fault, there is still value in trying to prevent dog bites through similar societal and policy mechanisms as those used for other types of injury prevention.

The study raises the question of what exactly are we trying to prevent? When dog aggression is described by canine ethologists, play is often excluded (Lockwood, 1995). Our participants appeared to echo these views, discussing the importance of “intention” of the dog when delivering a “bite”. However, any contact between skin and teeth can cause damage and require hospital treatment. In one study, 25% of dog bites to the head and neck of children were from “puppies” (Kasbekar et al., 2013), and puppies may be more likely to inflict such injuries during play than as a learned response to fear-inducing stimuli. Prevention initiatives that are designed to educate the public about behavioral signals are not suitable for preventing bites in contexts where these signals will not be displayed.

### Recommendations

Many dog experts believe the key to dog bite prevention is education regarding dog signalling and appropriate human behavior around the dog (Mills and Levine, 2006, Cornelissen and Hopster, 2010, Overall, 2010). The educational approach to prevention relies heavily on whether the victim believes that they require this knowledge, has access to the knowledge, can make sense of the knowledge and can implement their knowledge and take appropriate action to avoid the bite. This requires a recognized perceived need for engagement with education surrounding how to prevent a bite, because the person believes that a bite may happen to them, and is preventable. As these participants have demonstrated, this may not be the case. The “instantaneous” nature of bites was also recognized in all interviews. Many victims clearly did not have time to step back and assess the risk level immediately before the bite and this was a major factor leading to some bite incidents and despite their beliefs in hindsight, most could not really have been expected to behave differently at the time to prevent it.

Our findings suggest that prevention strategies could benefit from a focus on the ability of the victim to assess the risk in the immediacy of any situation with a dog and have clear instructions on what to do at that moment regardless of prior knowledge or experience with dogs. An education campaign portraying realistic and serious consequences, (“it could happen to me”—such as those used in injury prevention initiatives for other issues such as drink driving, not having a smoke alarm, chip fat fires), may be more effective than just telling people about dog body language and describing high risk situations. Participants also valued information about dogs gained from social contacts such as family and friends, an example of truth bias, suggesting that improving access to knowledge about dogs through social contacts such as face-to-face interactions with people who have been bitten, or even social media, may be more effective than more traditional educational programs delivered via media, books, DVD, or training courses. However, any such campaigns would need to be well-thought out and multifaceted to deal with varied contexts rather than a one-size-fits-all approach. They would also need to address issues of efficacy and the perceived level of threat, as both are required for behavior change (Peters et al., 2013).

A primary goal of injury prevention strategy should be to create environments where it is difficult for individuals to make mistakes or behave inappropriately (Hemenway, 2013). Many things had to happen for an injury to occur and it is ineffective to focus on the last like we do with dog bites. More consideration needs to be given to the contextual factors responsible for the circumstances and events that can lead to injury, as highlighted by the ecological approach (McClure, 2010). A sour study highlights, not all bites occur because a victim or owner behaved “wrongly,” and bites can occur in a number of different contexts that require separate consideration in terms of prevention. Furthermore, this contextual approach may help to identify ways in which society can live with dogs in a manner that causes the least possible harm.

Our findings highlight the importance of societal culture and education surrounding living with dogs, in particular regarding recognition and treatment of the early signs that a dog may bite in the future. This includes what to consider when acquiring a dog or puppy (Westgarth et al., 2012), as breeding, early experiences and ongoing socialization and training are thought to have significant effects on adult behavior (Appleby et al., 2002, Newman, 2012).

The second goal of injury prevention strategy is to ensure that when there is a mistake, nobody gets seriously injured (Hemenway, 2013). For example, seat belts do not stop car accidents from happening, but still reduce the level of injury. As aggression is part of the normal behavioral communication repertoire of dogs, and given the barriers to prevention we have described here, it may be impossible to prevent many dog bites in reality. An alternative approach would be for practitioners to focus on educating people about how to reduce the severity of bite injuries when they occur. Following on from our earlier example, it may be possible to reduce the effect of a dog bite by reducing the intensity or strength of the bite and thus the damage it inflicts. Reducing the intensity of bite damage would require an adjustment in educational strategy, away from preventing bites, toward increasing emphasis on training or breeding for “inhibited” bites and knowledge of how to behave during an attack. The latter approach has been used in some educational material for children, for example, advising standing still “like a tree” or rolling into a ball (Beatree.com, 2015, safetyarounddogs.org, 2015), but delivered in a context that is still primarily aimed at preventing the dog from wanting to bite at all rather than reducing the intensity of the injury. The importance of training bite inhibition in puppies has been previously recommended by some (Dunbar, 2001). However, the approach of selecting for dogs with inhibited bites may be controversial, for example the topic of Breed Specific Legislation concerning whether different breeds present variable risk in terms of intensity of damage caused by a bite even if there is no clear evidence of differences in risk of a bite occurring. There is also likely to be disagreement from people who likely feel that a person should not have to be exposed to any bite contact from a dog.

## Conclusions

Perceptions of why dogs bite and how they should be prevented are far more complex than first appear. Although more research is required, our findings suggest that the apparent instantaneous nature of bites and recognized psychological barriers to being receptive to educational intervention, may mean bites are not as easily preventable as previously assumed. The perception that “it would not happen to me” until a bite occurred, is a significant barrier to current prevention initiatives. Drawing from experience of other injury prevention contexts, a cultural change in the approach to dog bite prevention may be required. Rather than assigning fault to victims or owners and targeting “high-risk” individuals, the focus should be on intervention at the population level on creating a primary environment where dog bites are less likely to occur in the first place and minimizing damage caused when dogs do bite.

## Figures and Tables

**Table 1:** Dog-bite victim interview participant demographic data

Participant	Gender	Age	Education	Employment	Marital status	Current dog owner?
Annie	F	50s	O'level/GCSE	Retired	Married	Yes
Barbara	F	50s	Postgraduate	Professional	Single	Yes
Claire	F	20s	Degree	Retail	Single	Yes
Debra	F	50s	O'level/GCSE	Admin	Married	No
Ellie	F	20s	Postgraduate	Admin	Single	No
Fran	F	20s	NVQ	Dog groomer	Single	Yes
Gina	F	40s	O'level/GCSE	Admin	Single	No
Helen	F plus husband	30s	Postgraduate	Professional	Married	Yes

F, female; GCSE, General Certificate of Secondary Education; NVQ, National Vocational Qualification.

**Table 2:** Description of the main dog bite incident discussed for each dog bite victim participant

Participant	Bite from own dog?	Bite context	Reason given for bite	Type of main bite discussed	Bite location	Medical treatment sought?	Participant previously bitten?	Time lapse between specific injury and interview
Annie	No	Delivering newspapers to neighbors	*I think it's bored, I do. Because it has nothing to do, it's there all day and […] they don't take it out to walk. But let's face it, if you're stuck in a house all day you'd go mad wouldn't you. I would.*	Single	Leg	Yes—hospital tetanus	No	1 year
Barbara	No	Walking friend's dog, who had aggression towards other dogs	*It's what I would call redirected aggression. He couldn't get to what he was trying to get at, I pulled him back [using the lead], so I was the nearest thing that he could actually take it out on, and he did.*	Single	Leg	No	Yes	2 months
Claire	No	Family member's dog with neurologic problem, interrupting dog circling	*He started to circle which is something I'd seen him doing before, and my sister used to just go and stop him, kind of interrupt the behavior, and that would be it. I went to do that, in this instance, and whether it had got too far because he'd been going at it too long and also I didn't know him very well, and that's when he went for me.*	Multiple	Legs and chest	No	Yes	5 years
Debra	Yes (previous dog)	Multiple, handling dog (drying, grooming, touching, talking to)	*I would say she was pretty much the alpha, in a way […] She didn't want me to do what I was doing. And she'd already told me not to. But I carried on.*	Single on multiple occasions from same dog	Hands	No	Yes	Multiple over lifetime. Dog died 6 months previously.
Ellie	—	Running in a park	*I really have no idea why it bit me. I jokingly said “I think it sensed I was the weakest in the pack” (both laugh). But I'm sure it wasn't that intelligent […] maybe just cause I was running past it, it just though “ooh, rabbit” (chuckles).*	Single	Leg	No	Yes	1 month
Fran	No	Grooming dog	*As I said it's been a while since she was groomed so her coat was longer and probably a bit more tattier than usual and as I say she's out of sorts, getting older. Ailing, with her teeth, the nails all crossed, so I can only assume that she must have been in a bit of pain. And, and we just touched the wrong nerve. (laughter).*	Single	Arm	Yes—rang doctor, iodine patch	Yes	6 months
Gina	—	Delivering parcel to a neighbor's house	*[The other dog] was like a warning type, you know like a guard dog but without, it didn't want to hurt, it was just like, sort of like “you're on my territory” type of thing like you would expect a dog to do. But the other one was just vicious. You could see it was just vicious, and subsequently after I've been past this house again, it still has that same demeanor.*	Single	Leg	Yes—hospital tetanus	Yes	1 year
Helen	Yes	Kissing dog when asleep on settee	*It was because he was fast asleep, and I just saw him fast asleep there, looking all cute and cuddly, and obviously I had had a few glasses of wine and I thought “oh there's my little dog” I went up and (kiss noise) “Percy” and that obviously woke him up and startled him.*	Single	Face	Yes—walk in center, antibiotics	No	2 months

## Box 1 Semi-structured interview schedule for dog-bite victims

**Table:**

Interview schedule—dog bite victim experience	
*Bitten by own dog* 1. Tell me about your dog 2. Have you had other dogs before this dog?	*Bitten by someone else's dog* 1. Have you ever owned a dog? 2. Tell me about dogs that you have owned.
• Tell me about them 3. Have you ever been bitten by a dog before this bite? (How would you define a dog bite?) Tell me about this recent dog bite: Before 4. When did the bite occur? 5. Where were you? 6. What were you doing? 7. (If not own dog): • Did you previously know the dog? • Can you describe the dog? (History?) 8. What was the dog doing just before the bite? 9. What were you doing just before the bite? During 10. Can you describe the bite incident in detail to me? • Could you tell me how you thought the dog's demeanor was just before the bite? • Did you approach the dog or did it approach you? • What was the purpose of the interaction with the dog around the time of the bite? • Where did it bite you, how many times and how deeply? 11. Why do you think that the dog bit you? 12. What makes you think that? • Where do you get information about dogs from? • Do you think that the bite was intentional? After 13. Can you describe what damage the bite did to you? 14. How did you react after the bite? 15. (If not own dog) How did the owner react? 16. How did that make you feel? 17. Did you need medical help after the bite? If so what did you do? How did the bite affect the rest of your day? 18. Has the bite affected you? • Physically? • Mentally? 19. Do you think that anybody is to blame for the bite happening? If so, who? 20. If you could go back to the day of the dog bite, is there anything that you would change? 21. Are you planning to do anything in the future to prevent another bite from happening? 22. Has being bitten changed how you feel about dogs?	
